# Children's exposure to second-hand smoke 10 years on from smoke-free legislation in England: Cotinine data from the Health Survey for England 1998-2018

**DOI:** 10.1016/j.lanepe.2022.100315

**Published:** 2022-02-03

**Authors:** Harry Tattan-Birch, Martin J. Jarvis

**Affiliations:** Department of Behavioural Science & Health, University College London, London, UK

**Keywords:** Passive smoking, Cotinine, Children, Smoke-free, Smoking ban

## Abstract

**Background:**

We aimed to investigate trends in children's exposure to second-hand tobacco smoke in England from 1998 to 2018.

**Methods:**

We used twenty-one years of data from the Health Survey for England, a yearly repeated cross-sectional population study. A total of 49,460 children participated between 1998 and 2018, of whom 17,463 were biochemically confirmed non-smokers aged 4-15. We examined changes in (i) the proportion of children living in reported smoke-free homes and (ii) second-hand smoke uptake, measured quantitatively using saliva cotinine concentration.

**Findings:**

The percentage of children living in a home reported to be smoke-free increased from 63.0% (95% CI 60.5%-65.2%) in 1998 to 93.3% (91.8%-94.6%) in 2018. This increase was most pronounced among children with a smoker parent, rising from 17.1% (14.7%-19.8%) to 75.9% (70.8%-80.4%). Segmented regression showed that the rate of adoption of smoke-free homes accelerated leading up to the 2007 ban on smoking in public places, growing most rapidly in the four years after its entry into law. Between 1998 and 2018, there was a ten-fold decline in geometric mean cotinine among non-smoking children, from 0.50 ng/ml (0.46-0.56) to 0.05 ng/ml (0.04-0.06). A total of 65.0% (61.2%-68.6%) of children had undetectable cotinine in 2018, up from 14.3% (12.7%-16.0%) in 1998. Children living in rented accommodation were more exposed than those from owner-occupied households, but they experienced similar relative declines across years.

**Interpretation:**

Cotinine data show that children's exposure to second-hand smoke has fallen by some 90% since 1998, with an apparent acceleration in adoption of smoke-free homes since the 2007 ban on smoking in public places. A norm has emerged that sees smoking in the home as inappropriate, almost universally where parents are non-smokers, but also increasingly among smoking parents.

**Funding:**

Public Health England, now the Office for Health Improvement and Disparities, and Cancer Research UK.

**Research in context**

**Evidence before this study**

We searched PubMed up to October 1^st^ 2021 for papers on children's exposure to second-hand smoke in England, using the terms (“passive smok*” OR “second-hand smok*” OR “secondhand smok*”) AND “England” AND “Children”. Previous studies showed that children's exposure to second-hand smoke had been declining in England since the 1980s, with saliva cotinine used as an objective marker of smoke uptake. As exposure fell, a growing proportion of children surveyed had cotinine concentrations below the limit of detection, yet none of the relevant studies used statistical methods that properly account for these undetectable values — with most setting undetectable values equal to half the limit of detection and thus overestimating average cotinine levels. Studies also reported on a growth in the adoption of smoke-free homes over time, with early evidence suggesting the rise continued after legislation banning smoking inside public places was introduced in 2007. No data were reported beyond 2012.

**Added value of this study**

We present two decades of data on children's exposure to second-hand smoke in England from 1998 to 2018, a period which saw the introduction of legislation banning smoking inside public places. Unlike previous reports, tobit regression was used to properly account for undetectable cotinine values, and trends were modelled using linear and restricted cubic splines.

**Implications of all the available evidence**

There has been close to a twenty-fold reduction in children's exposure to second-hand smoke in England since the late 1980s, with an apparent acceleration in progress after legislation mandating smoke-free public places was introduced in 2007. Now more than 90 percent of children overall, including 75 percent of children with smoker parents, live in a home reported to be smoke-free, an occurrence that was once rare. The near elimination of exposure in the home may now be a realistic target for policy in England.

Alt-text: Unlabelled box

## Introduction

The issue of second-hand tobacco smoking in the home first attracted widespread attention in 1981, with Hirayama's report of raised lung cancer risks in non-smoking Japanese women with husbands who smoke.^1^ Cotinine-based surveys in the UK soon showed high levels of exposure in children with smoking parents,^2^^,^^3^ as well as adverse effects on their health.^4^ There was mounting concern over the need to protect children from other people's smoke. Already, by the turn of the millennium, children's exposure in the home had begun a secular decline^5^ as parental smoking fell, and there were increasing moves to restrict smoking in enclosed public places such as bars, restaurants and workplaces.^6^^,^^7^ Concerns were raised that implementation of legislation mandating smoke-free public places in 2007 might drive smoking back into the home.^8^ Early evidence suggested that, contrary to these speculations, children's exposure to smoking in the home continued to fall in the years directly following its entry into law.^9^ Similar declines in second-hand exposure occurred across other countries — including Germany, the US, Scotland, and South Korea — and there was growing acceptance of norms against smoking inside the home, including among parents who were themselves smokers.^10^, ^11^, ^12^, ^13^ In 2010 the then UK government included in its national tobacco control plan^14^ the ambition to see two-thirds of households with smoking parents go smoke-free by 2020.

The aim of this report, extending on previous publications,^9^^,^^15^^,^^16^ is to investigate current trends in children's exposure to second-hand smoke, a decade after the implementation of smoke-free legislation in England. We use twenty years of saliva cotinine data, an objective biomarker of nicotine uptake, collected between 1998 and 2018 from the Health Survey for England. We also detail the percentage of children who live in homes reported to be smoke-free, testing whether the rate of adoption of smoke-free homes changed over time — through the years leading up to and following the 2007 ban on smoking in public places. Finally, we present how trends in exposure differ by children's socio-economic background, examining whether historic inequalities have narrowed or widened.

## Methods

### Setting

The Health Survey for England is an annual cross-sectional survey that is designed to provide representative samples of households in England across key demographic variables including sex, age, location, and socio-economic background. It uses a clustered, stratified multi-stage sample design. An additional boost sample was recruited from specific demographic groups in some years. Full details, including yearly reviews by Research Ethics Committees, are available in published reports and online. In participating households, every adult and up to two children are interviewed in their home. In homes with three or more children, two children are selected at random for interview. From 2015 onwards, up to four children could be interviewed. Non-response weights (described below) account for this under sampling of children from large households. This is followed by a visit from a nurse approximately one week later, when biological measurements including saliva samples are collected. 59% of eligible households participated in 2018, with 91% of children in these participating homes being interviewed, 54% seeing a nurse, and 35% providing saliva.^17^ Response rates have declined over time and are much lower than in 1998, when 74% of eligible households participated, with 96% of children in these participating homes being interviewed, 83% seeing a nurse, and 81% providing saliva.^18^

### Measurements

Parental smoking was determined at the initial interview, with adult smokers identified as those responding “yes” to the question “Do you smoke cigarettes at all nowadays?”. To encourage more accurate self-report, children aged 8 and above were asked about smoking through a self-completion booklet with a six-item scale: “I have never smoked”; “I have only smoked once or twice”; “I used to smoke sometimes, but I never smoke a cigarette now”; “I sometimes, smoke, but I don't smoke every week”; “I smoke between one and six cigarettes a week”; and “I smoke more than six cigarettes a week”. The latter three responses indicated that a child was a smoker. Children aged under 8 were not asked about their smoking status, and they are assumed to be non-smoking unless their saliva cotinine concentration exceeded 12ng/ml.

Presence of smoking inside the home was characterised for an entire household based on responses from a single adult during the initial interview. The household reference person or their partner was asked "Does anyone smoke inside this house/flat on most days?". We define homes as smoke-free if the response to this question was "no", as in previous publications,^9^^,^^15^ although it is in principle possible that such homes could be mostly but not completely smoke-free. Children's socio-economic background was indexed by housing tenure of the household (owner-occupied/rented), as previous work has shown living in rented or social housing to be strongly associated with rates of adult cigarette smoking.^19^ Homes where the household reference person reported buying it with the help of a mortgage/loan or paying part rent and part mortgage were also considered “owner-occupied”. Homes where the reference person reporting squatting or living rent-free (<1% of sample) were excluded from the analyses by housing tenure.

### Cotinine

Saliva cotinine concentration is a sensitive and specific marker of recent nicotine intake over the past few days. It is widely accepted as the best biological indicator of second-hand smoke exposure.^20^ While there was no self-report from questionnaires documenting use of electronic cigarettes (“e-cigarettes”) in the home, cotinine measures would capture any second-hand exposure to nicotine e-cigarette aerosol. Saliva specimens were collected from children using dental roll or a straw before being sent to a laboratory to be assayed. Because saliva cotinine is highly stable, even at room temperature, any delay in the assay would have a negligible effect on results.^21^

An assay using liquid extraction and gas chromatography with nitrogen phosphorous detection (GC-NPD) was employed throughout 1998 and 2007.^22^ A new technique was introduced during 2008, using high-performance liquid chromatography coupled with mass spectrometry with multiple reaction monitoring (LC-MS/MS).^23^ Cross-validation showed the results from the two methods to be interchangeable, and regular quality controls were run to ensure reliability.^24^ The limit of detection was 0.1ng/ml.

### Samples

We used all data available from children aged 0-15 and their parents, collected between 1998 and 2018 inclusive. To estimate the proportion of children with smoking parents, the denominator used was all children aged 0-15 (N = 49,460) regardless of whether they smoked or provided a saliva sample. The sample used for cotinine-based results was confirmed non-smoking children aged 4-15 with saliva cotinine available (N = 17,463).

Children's cotinine samples were not collected in 2000. This year was therefore excluded from cotinine-based analyses, as were cotinine data from 1999 and 2004 when the nurse visit was only offered to ethnic minorities. Children were defined as non-smoking if they reported no current smoking and had cotinine levels under 12ng/ml, a cut-point identified as optimal for detecting active smoking.^25^ This cotinine cut-point also removed children with raised cotinine from use of e-cigarettes from the sample. As mentioned above, all children aged under 8 were considered non-smokers unless their cotinine levels were above 12ng/ml (<1% exceeded this threshold).

### Statistical analysis

All results in tables, with the exception of geometric mean cotinine, were calculated in SPSS 26, using the complex samples procedures to appropriately account for clustering and stratification in the survey design. Geometric mean cotinine and results in figures were calculated using the ‘survey’ package in R version 4.03, which provides analogous adjustments for survey design.^26^ Weights that adjust for non-response to the nurse visit were supplied from 2003 onwards. Further weights were made available in 2007, which accounted for non-participation in the saliva sample. We used these weights for all years where they were available. Our analyses were closely based on previous publications,^16^ but as they were not formally pre-registered, should be considered exploratory. We used an alpha of .05 and reported estimates alongside 95% confidence intervals (95% CIs).

The distribution of cotinine among non-smokers is positively skewed, which means it is most appropriately analysed using geometric rather than arithmetic mean.^27^ This requires a method to account for samples below the limit of detection (0.1ng/ml). Such samples have previously been imputed as having a value of half this limit (0.05ng/ml).^16^ This was a good approximation when only a small proportion of samples fell below the limit, but it has become increasingly inaccurate as second-hand smoke exposure has fallen — leading to substantial overestimates in geometric mean cotinine.^28^ Therefore, we instead used log-normal tobit regression, which is recommended when a large proportions of samples fall below the limit of detection.^29^ This models residuals in log cotinine as being drawn from a normal distribution where all values under the limit of detection (<0.1ng/ml) are censored.

We examined trends over time in (i) smoke-free homes and (ii) geometric mean cotinine among non-smoking children by parental smoking. These trends were modelled using logistic regression, for the former, and log-normal tobit regression, for the latter. To allow for flexible non-linear trends, year was transformed using natural splines with five knots (three breakpoints) placed at quantiles of the data. Splines are preferred over polynomials because trends rarely conform to linear, quadratic or cubic relationships.^30^ They are also preferred over categorisation, which neglects that outcomes are more similar in adjacent than distant years.^31^

Finally, we tested whether the rate at which smoker parents adopted smoke-free homes changed over time, in the years leading up to and following the 2007 ban on smoking inside public places. Our outcome was the percentage of children with smoking parents who were exposed to smoking inside the home. Declines in this outcome over time indicated increases in the adoption of smoke-free homes. Two different methods were used to model and test changes in trends. Firstly, we used linear splines (i.e., segmented log-binomial regression) with breakpoints in 2004, 2007, and 2010. This allowed us to estimate the average rate of change in exposure across four time periods: (i) from 1998-2004, (ii) during intense public debate of smoke-free legislation from 2004-2007, (iii) in the three years following its enactment into law from 2007-2010, and (iv) from 2010-2018. We also report the log-binomial regression coefficient, *B*, representing the relative rate of decline for each period compared with the previous one. A *B* that is positive indicates the rate of decline slowed, zero indicates it stayed constant, and negative indicates it accelerated. Secondly, we used natural cubic splines in logistic regression (as described previously), which allowed us to estimate the rate at which exposure changed across every time-point between 1998 to 2018. To do this, we used numerical differentiation on fitted values from the logistic regression, with finite differences of one thousandth of a year. Results from both approaches were displayed in a graph, with 95% CIs constructed using bootstrapping (percentiles from 1000 resampled cases).^32^

### Role of the funding source

The design of the study was informed by conversations with individuals at Public Health England, but they and Cancer Research UK had no role in the data analysis, writing of the report, or in the decision to submit the paper for publication.

## Results

The percentage of children (0-15 years) who had a parent that reported currently smoking cigarettes fell steadily over time, down from 41% in 1998 to 25.2% in 2018 (Table 1, left). A similar decline in parental smoking occurred among the subsample of children (4-15 years) with saliva cotinine levels that confirmed they were themselves non-smokers (Table 1, right).

**Table 1:** Left: Percentage of all children (aged 0-15) who have at least one parent that smokes cigarettes; Right: In the sample of confirmed non-smoking children (aged 4-15), percentage who have a parent that smokes, geometric mean cotinine, and 95^th^ percentile cotinine concentration.

	In all children aged 0-15		In confirmed non-smoking children aged 4-15			
	N	One or both parents smoke, % (95%CI)	N	One or both parents smoke, % (95%CI)	Geometric mean cotinine, ng/ml (95% CI)	95^th^ percentile cotinine, ng/ml
1998	3638	41.0 (38.8–43.1)	2095	39.3 (36.7–42.0)	0.50 (0.46–0.56)	5.70
1999	1841	39.6 (36.6–42.7)				
2000	1932	39.4 (36.5–42.4)				
2001	3934	39.6 (37.5–41.6)	1798	36.4 (33.7–39.2)	0.46 (0.42–0.50)	4.80
2002	2668	38.1 (35.5–40.7)	1064	37.8 (34.4–41.3)	0.41 (0.36–0.46)	4.80
2003	3667	39.0 (36.4–41.6)	1643	37.5 (34.0–41.2)	0.45(0.41–0.50)	5.20
2004	1650	33.0 (29.8–36.3)				
2005	1834	36.0 (33.0–39.2)	706	35.7 (30.9–40.8)	0.36 (0.31–0.42)	4.90
2006	3441	35.4 (33.1–37.7)	1411	33.8 (30.5–37.4)	0.20 (0.18–0.23)	3.70
2007	1727	33.5 (30.5–36.7)	695	31.4 (27.0–36.2)	0.16 (0.13–0.19)	4.30
2008	3439	33.9 (31.8–36.0)	1417	33.1 (30.3–35.7)	0.17 (0.15–0.19)	3.50
2009	1147	30.2 (26.4–34.2)	473	26.3 (21.6–31.5)	0.10 (0.08–0.13)	3.30
2010	2071	28.2 (25.6–31.0)	700	26.7 (23.0–30.8)	0.08 (0.07–0.10)	2.70
2011	2006	29.9 (27.0–32.9)	670	29.3 (24.8–34.4)	0.08 (0.07–0.10)	2.60
2012	2043	27.8 (25.2–30.5)	655	25.7 (21.4–30.5)	0.06 (0.05–0.07)	2.50
2013	2185	27.3 (24.7–30.0)	796	22.3 (19.2–25.8)	0.08 (0.07–0.10)	1.90
2014	2003	27.9 (25.1–30.8)	701	27.1 (23.2–31.5)	0.06 (0.05–0.08)	2.00
2015	2121	25.2 (22.8–27.8)	725	22.9 (19.5–26.6)	0.06 (0.05–0.08)	1.80
2016	2056	19.5 (17.1–22.2)	609	20.3 (16.8–24.2)	0.06 (0.05–0.07)	1.80
2017	1985	23.5 (21.1–26.1)	661	21.3 (18.2–24.9)	0.06 (0.05–0.07)	1.80
2018	2072	25.2 (22.8–27.8)	644	21.7 (18.2–25.5)	0.05 (0.04–0.06)	1.10

95%CI = 95% confidence interval.

**Figure 1:**
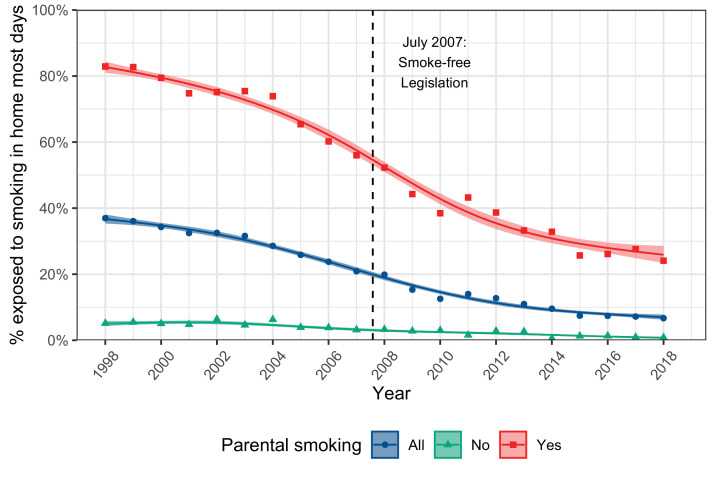
**Percentage of children (0-15 years) in England exposed to smoking inside the home most days by parental smoking, 1998-2018.** Solid lines represent fitted values from logistic regression, with trends modelled using natural cubic splines. Shaded bands represent 95% CIs. Points show estimates from each yearly survey wave (available with confidence intervals in Supplementary Table 1).

The proportion of children living in a home reported to be smoke-free rose substantially between 1998 and 2018, from 63.0% to 93.3% (Figure 1). This was partially explained by falling parental smoking rates. However, Figure 1 shows that it was also attributable to the growing proportion of homes with smoking parents reporting no smoking in the home most days (up from 17.1% in 1998 to 75.9% by 2018).

Adoption of smoke-free homes varied over time. Supplementary Figure 1 shows the yearly rate of decline in exposure to smoking in the home among children with smoker parents, using two different methods to model trends. Analyses with linear splines (i.e., segmented regression) showed that the relative rate of decline in exposure accelerated from an average of 2.3% per year between 1998-2004 to 7.7% between 2004-2007 — a period which saw intense national debate surrounding proposed smoke-free legislation (*B* = -0.057, 95% CI = -0.078 to -0.036, *p* < .00001). This decline continued at a rate of 9.7% per year from 2007-2010, following the enactment of smoke-free legislation (*B* = -0.023, 95% CI = -0.062 to 0.017, *p* = .26), before slowing to 6.4% between 2010-2018 (*B* = 0.036, 95% CI = -0.001 to 0.073, *p* = .06). More granular analyses with natural cubic splines produced similar results, showing that the yearly rate of decline gradually sped up from around 2% in the early 2000s to a peak of 9.9% in 2010 (Supplementary Figure 1).

**Figure 2:**
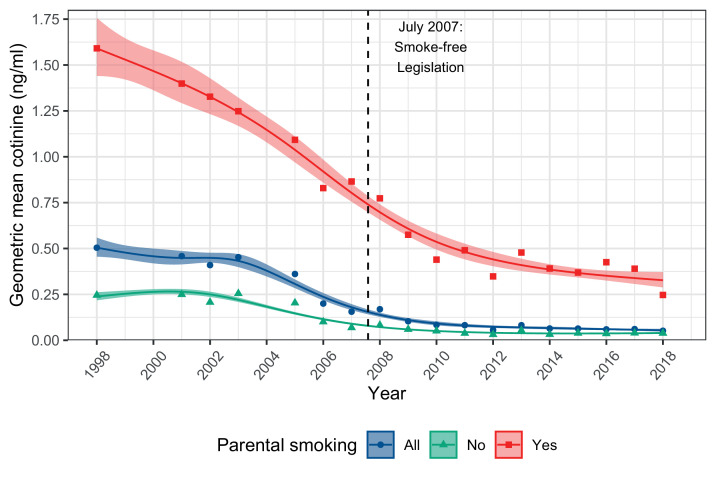
**Geometric mean cotinine among non-smoking children (4-15 years) in England by parental smoking, 1998-2018.** Solid lines represent fitted values from log-normal tobit regression, with trends modelled using natural cubic splines. Cotinine values below 0.1ng/ml were undetectable by the assay. Shaded bands represent 95% CIs, and points show estimates from each yearly survey wave.

Cotinine data confirmed the substantial decline in children's second-hand smoke exposure over time. Geometric mean cotinine in verified non-smoking children fell by 90% between 1998 and 2018, from 0.50ng/ml to 0.05 ng/ml (Table 1, right). Figure 2 shows that falls were most pronounced among children whose parents smoke, mirroring declines in reported exposure to smoking inside the home most days.

Observed cotinine values also validated reports of homes being smoke-free; across every year, children with smoking parents living in homes that were reported smoke-free had far lower cotinine concentrations than those in homes that were not (Table 2, Supplementary Figure 2). Supplementary Table 2 shows the percentage of children with undetectable cotinine by parental smoking and whether or not the home was smoke-free. In 1998, 21% of children whose parents did not smoke had undetectable cotinine, compared with only 3.3% of those with smoking parents. By 2018, the corresponding figures had reached 75.3% and 27.5%.

**Table 2:** Geometric mean cotinine in confirmed non-smoking children (4-15 years) by parental smoking and whether or not home is reported as smoke-free, 1998-2018.

	Geometric mean cotinine, ng/ml (95% CI)								
	No smoking in home most days			Smoking in home most days			All		
	No parental smoking	One or both parents smoke	All	No parental smoking	One or both parents smoke	All	No parental smoking	One or both parents smoke	All
1998	0.24 (0.21–0.26)	0.47 (0.36–0.60)	0.25 (0.23–0.27)	0.67 (0.46–0.98)	1.99 (1.82–2.18)	1.84 (1.67–2.02)	0.25 (0.22–0.27)	1.59 (1.44–1.76)	0.50 (0.46–0.56)
2001	0.24 (0.22–0.26)	0.44 (0.35–0.56)	0.25 (0.23–0.27)	0.79 (0.55–1.13)	1.85 (1.67–2.05)	1.74 (1.57–1.93)	0.25 (0.23–0.27)	1.40 (1.25–1.56)	0.46 (0.42–0.50)
2002	0.19 (0.17–0.22)	0.34 (0.25–0.46)	0.21 (0.18–0.23)	0.88 (0.54–1.42)	1.97 (1.73–2.24)	1.83 (1.61–2.08)	0.21 (0.18–0.24)	1.33 (1.14–1.54)	0.41 (0.36–0.46)
2003	0.24 (0.22–0.26)	0.40 (0.33–0.49)	0.25 (0.23–0.28)	1.09 (0.75–1.60)	1.77 (1.59–1.98)	1.70 (1.52–1.89)	0.25 (0.23–0.28)	1.25 (1.12–1.40)	0.45 (0.41–0.50)
2005	0.20 (0.18–0.22)	0.46 (0.46–0.47)	0.22 (0.20–0.24)	0.65 (0.64–0.65)	1.60 (1.44–1.79)	1.49 (1.34–1.66)	0.20 (0.19–0.22)	1.09 (1.00–1.19)	0.36 (0.31–0.42)
2006	0.10 (0.09–0.11)	0.31 (0.28–0.35)	0.12 (0.10–0.13)	0.59 (0.59–0.60)	1.40 (1.29–1.53)	1.32 (1.22–1.43)	0.10 (0.09–0.11)	0.83 (0.75–0.91)	0.20 (0.18–0.23)
2007	0.07 (0.06–0.08)	0.30 (0.19–0.46)	0.08 (0.07–0.10)	0.32 (0.13–0.82)	1.55 (1.21–1.99)	1.41 (1.09–1.82)	0.07 (0.06–0.08)	0.87 (0.67–1.12)	0.16 (0.13–0.19)
2008	0.08 (0.07–0.08)	0.35 (0.33–0.36)	0.10 (0.10–0.11)	0.52 (0.52–0.52)	1.60 (1.47–1.73)	1.39 (1.29–1.51)	0.08 (0.08–0.09)	0.77 (0.71–0.84)	0.17 (0.15–0.19)
2009	0.05 (0.04–0.06)	0.24 (0.19–0.30)	0.07 (0.05–0.08)	1.14 (1.14–1.15)	1.47 (1.31–1.65)	1.40 (1.19–1.64)	0.06 (0.05–0.07)	0.57 (0.47–0.70)	0.10 (0.08–0.13)
2010	0.04 (0.04–0.05)	0.22 (0.17–0.29)	0.06 (0.05–0.07)	1.22 (1.22–1.22)	1.11 (0.90–1.38)	1.13 (0.95–1.35)	0.05 (0.04–0.06)	0.44 (0.35–0.54)	0.08 (0.07–0.10)
2011	0.04 (0.03–0.04)	0.23 (0.20–0.27)	0.06 (0.05–0.07)	0.96 (0.96–0.96)	1.34 (0.81–2.23)	1.31 (0.82–2.08)	0.04 (0.03–0.05)	0.49 (0.38–0.63)	0.08 (0.07–0.10)
2012	0.03 (0.03–0.04)	0.19 (0.14–0.26)	0.04 (0.04–0.05)	0.43 (0.42–0.44)	0.83 (0.61–1.13)	0.76 (0.58–1.00)	0.03 (0.03–0.04)	0.35 (0.28–0.43)	0.06 (0.05–0.07)
2013	0.05 (0.04–0.06)	0.26 (0.24–0.30)	0.06 (0.05–0.08)	0.81 (0.77–0.84)	1.04 (0.83–1.30)	0.99 (0.82–1.21)	0.05 (0.04–0.06)	0.48 (0.40–0.57)	0.08 (0.07–0.10)
2014	0.03 (0.03–0.04)	0.22 (0.18–0.26)	0.05 (0.04–0.06)	2.50 (2.50–2.50)	1.02 (0.81–1.28)	1.02 (0.81–1.28)	0.03 (0.03–0.04)	0.39 (0.33–0.47)	0.06 (0.05–0.08)
2015	0.04 (0.03–0.05)	0.22 (0.18–0.27)	0.06 (0.05–0.07)	0.17 (0.17–0.17)	1.28 (1.15–1.43)	1.07 (0.94–1.22)	0.04 (0.03–0.05)	0.37 (0.31–0.44)	0.06 (0.05–0.08)
2016	0.04 (0.03–0.04)	0.24 (0.19–0.30)	0.05 (0.04–0.06)	0.85 (0.84–0.85)	1.31 (0.93–1.83)	1.25 (0.92–1.71)	0.04 (0.03–0.04)	0.43 (0.34–0.53)	0.06 (0.05–0.07)
2017	0.04 (0.03–0.05)	0.22 (0.18–0.27)	0.05 (0.05–0.06)	0.33 (0.33–0.33)	1.23 (1.10–1.36)	1.13 (1.03–1.25)	0.04 (0.03–0.05)	0.39 (0.34–0.45)	0.06 (0.05–0.07)
2018	0.04 (0.03–0.05)	0.15 (0.13–0.17)	0.05 (0.04–0.06)	0.00 (0.00–0.00)	0.76 (0.70–0.83)	0.69 (0.64–0.75)	0.04 (0.03–0.05)	0.25 (0.20–0.30)	0.05 (0.04–0.06)

95%CI = 95% confidence interval.

**Figure 3:**
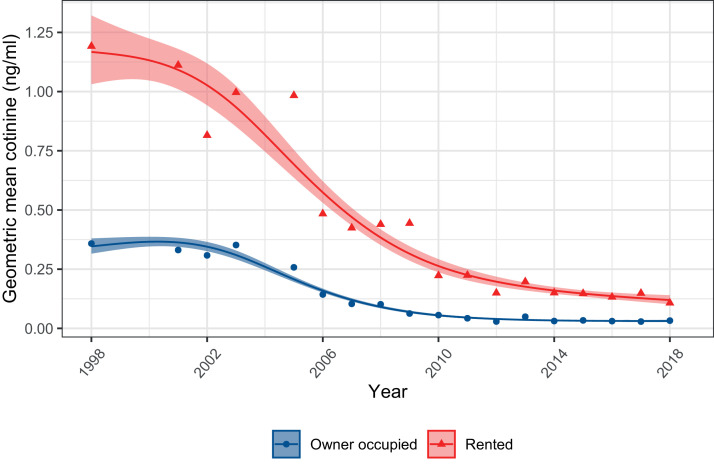
**Geometric mean cotinine among non-smoking children (4-15 years) in England by housing tenure, 1998-2018.** Solid lines represent fitted values from log-normal tobit regression with trends modelled using natural cubic splines. Cotinine values below 0.1ng/ml were undetectable by the assay. Shaded bands represent 95% CIs, and points show estimates from each yearly survey wave.

Table 3 shows inequalities in children's exposure over time by housing tenure. Children from owner-occupied households had significantly lower rates of parental smoking than those from rented accommodation. Relative declines in parental smoking were similar across both groups, but absolute declines were much greater in children from rented households (30.6% versus 61.0% in 1998, declining to 14.1% versus 33.5% in 2018). In 1998, homes with smoking parents that were owner-occupied were more than twice as likely to be smoke-free as those that were rented (22.1% versus 7.9%). However, adoption of smoke-free homes rose rapidly over time across both groups such that, by 2018, there was a substantial attenuation of this disparity (73.5% versus 69.5%). Similar trends were observed for cotinine measures. Figure 3 shows that, from 1998 to 2018, there was a ten-fold decrease in geometric mean cotinine among verified non-smoking children from both groups, leading to a sharp reduction in absolute inequalities. Relative inequalities nonetheless persisted; geometric mean cotinine remained over two times higher in children from rented compared with owner-occupied homes across all survey years (1.19ng/ml versus 0.36ng/ml in 1998; 0.11 ng/ml versus 0.03ng/ml in 2018; Supplementary Table 3).

**Table 3:** Differences by housing tenure in the percentage of confirmed non-smoking children (aged 4-15) in England with a smoker parent (Left) and living in a smoke-free home (Right), from 1998-2018.

	One or both parents smoke, % (95%CI)		Smoke-free home, % (95%CI)			
	All		No parental smoking		One or both parents’ smoke	
	Owner-occupied	Rented	Owner-occupied	Rented	Owner-occupied	Rented
1998	30.6 (28-33)	61.0 (57-65)	95.6 (94-97)	94.9 (91-97)	22.1 (19-26)	7.9 (6-11)
2001	27.6 (25-30)	60.0 (41-48)	96.7 (95-98)	95.3 (91-98)	27.4 (23-32)	10.8 (8-15)
2002	32.5 (29-36)	50.3 (45-56)	96.0 (94-97)	93.7 (89-97)	27.3 (22-33)	16.3 (11-23)
2003	30.1 (28-33)	59.3 (55-64)	96.4 (95-97)	91.1 (88-94)	33,1 (30-37)	14.6 (12-18)
2005	29.0 (26-32)	54.9 (50-60)	97.7 (96-99)	96.1 (94-98)	49.2 (44-55)	17.0 (13-22)
2006	26.3 (24-29)	52.5 (48-57)	98.5 (98-99)	93.9 (89-97)	42.4 (37-.48)	26.7 (21-34)
2007	22.4 (20-25)	56.0 (51-61)	99.1 (99-99)	93.2 (89-96)	41.9 (36-48)	29.4 (23-37)
2008	20.8 (18-24)	56.0 (51-61)	97.3 (96-98)	93.3 (90-96)	54.1 (47-61)	43.9 (37-51)
2009	19.9 (15-25)	45.5 (34-58)	98.3 (95-99)	83.5 (68-92)	69.1 (54-81)	30.1 (19-45)
2010	18.9 (15-24)	42.5 (34-51)	98.2 (96-99)	89.9 (83-94)	67.5 (56-77)	51.4 (39-64)
2011	19.1 (14-25)	46.3 (38-55)	100	94.5 (90-97)	61.4 (44-76)	56.8 (45-68)
2012	18.9 (15-24)	37.3 (30-45)	98.8 (97-100)	95.9 (93-98)	56.5 (41-71)	62.5 (51-73)
2013	13.8 (10-18)	35.9 (30-42)	98.0 (96-99)	96.2 (92-98)	63.2 (46-77)	55.5 (45-66)
2014	18.4 (14-24)	38.3 (32-45)	100	99.8 (98-100)	66.5 (53-78)	62.8 (52-73)
2015	15.8 (12-20)	36.5 (30-44)	98.9 (94-100)	99.5 (99-100)	80.8 (67-90)	66.6 (54-77)
2016	12.7 (10-17)	32.8 (26-40)	99.0 (99-99)	99.1 (96-100)	80.7 (66-90)	62.5 (49-74)
2017	11.7 (9-15)	34.6 (29-41)	99.8 (98-100)	98.8 (97-99)	81.1 (64-91)	62.1 (51-72)
2018	14.1 (11-18)	33.5 (27-41)	99.8 (99-100)	99.7 (99-100)	73.5 (61-83)	69.5 (58-79)

95%CI = 95% confidence interval.

## Discussion

There has been a dramatic decline in children's exposure to second-hand smoke in England over the past two decades, with cotinine levels falling by 90% between 1998 and 2018. The decline has been driven partly by a reduction in parental cigarette smoking — down from 41.0% in 1998 to 25.2% in 2018 — but also by an emerging trend that has seen parents who continue to smoke adopt a policy of no smoking within the home (17.3% in 1998, 61.3% in 2012, 75.9% in 2018). Saliva cotinine measures served both to validate the significance of such smoke-free home policies for lowering children's exposure and to quantify the extent of their overall second-hand smoke intake. We found that the years preceding and following the 2007 ban on smoking in public place were associated with an acceleration in the rate of decline in children's exposure to second-hand smoke, contradicting speculation that smoke-free legislation would drive smoking back into the home. Similar major declines in exposure to second-hand smoke have been observed across many countries throughout the globe.^10^, ^11^, ^12^, ^13^^,^^28^^,^^33^

Concerns have been raised that falls in second-hand smoke exposure may occur more slowly among children from disadvantaged backgrounds, leading to a widening in social inequalities. A recent report from the charity Action on Smoking and Health (ASH) cited exposure in social housing as an important problem to monitor.^34^ The majority of renters in the UK are in social housing or supported by government housing benefit/allowance.^35^ Therefore, our results may help to partly alleviate these concerns. While we found that cotinine levels were higher among children living in rented than owner-occupied households, exposure declined at a similar relative rate across both groups. By 2018, adoption of smoke-free homes by smoking parents in rented households (69.5%) had almost reached parity with owner-occupiers (73.5%), both representing substantial increases from 1998. While absolute disparities in exposure to second-hand smoke have been greatly reduced, social inequalities can still persist through the intergenerational transmission of smoking, whereby children whose parents smoke have an increased risk of themselves becoming smokers.^36^ Moreover, our data cannot rule out the possibility that notable inequalities in exposure remain between social and private renters, such as those caused by the lower prevalence of home no-smoking rules in social housing.^37^ There are many facets of social disadvantage, most of which raise a child's risk of second-hand exposure.^6^ Here we focus on housing tenure, because of its close relation to exposure in the home and its strength as a predictor of smoking in England.^19^ Further research examining trends across other socio-economic variables would nonetheless be valuable.

The percentage of children with living in smoke-free homes has risen continuously over the past two decades, but this trend has not been linear. Time-series analysis showed there was an acceleration in the rate of adoption of smoke-free homes beginning at the time of intense national debate surrounding the proposed ban on smoking in public places, continuing for some years after its entry into law in 2007 (Figure 1). This suggests that the ban on smoking in public places, despite not extending to smoking within the home, was an important factor contributing to a general denormalization of smoking in enclosed spaces, which in turn prompted more homes to become smoke-free.

Our study has several strengths. It uses two decades of data from large, representative samples of children and their parents. Because of this large sample size, we were able to precisely estimate trends across years using logistic and log-normal tobit regression. The smoking status of parents and children was determined through self-report, with saliva cotinine used to biochemically confirm reported non-smoking in children. Cotinine data also allowed us to validate that children living in homes reported to be smoke-free had substantially lower intake of second-hand smoke. A limitation of this study was the decline in response rates over time, with a falling percentage of children giving saliva. This trend towards lower participation rates has occurred across most major surveys in recent years, but we have attempted to limit the impact on findings through weighting adjustments for non-participation and non-provision of saliva samples. It is also possible that some of the apparent narrowing of (absolute) disparities between children in rented and owner-occupied homes is confounded by changes in characteristics of families who rent — such as those caused by the falling affordability of homes.^38^ Recent data shows that, despite these changes, housing tenure remains a powerful determinant of smoking-related inequalities in England.^19^ Finally, while there was no questionnaire measure of e-cigarette use in the home, cotinine values captured any nicotine intake from second-hand e-cigarette aerosol.

The past two decades have witnessed an exceptional decline in children's exposure to second-hand smoke in England, with the emergence of a social norm that has seen a growing proportion of parents choosing not to smoke inside the home. Now two-thirds of homes containing children with smoking parents are smoke-free, and children's cotinine levels are a tenth of what they were twenty years ago. Moreover, counter to fears that legislation establishing smoke-free public places would displace smoking back into the home, it has instead been accompanied by an accelerated rate of adoption of smoke-free homes. Elimination of children's exposure to second-hand smoke at home as a major public health issue is now a realistic target for policy in England.

## Declaration of interests

The authors have no interests to declare.

## Appendix Supplementary materials

Image, application 1
