# Supplementary material for: Children's exposure to second-hand smoke 10 years on from smoke-free legislation in England: Cotinine data from the Health Survey for England 1998-2018
Source: Lancet Reg Health Eur. 2022 Feb 3;15:100315. doi: 10.1016/j.lanepe.2022.100315 (PMC8819129; doi:10.1016/j.lanepe.2022.100315)
Supplement: Supplementary file 1 [file mmc1.docx]

**Supplementary Table 1: Percentage of children (0-15 years) living in a smoke-free home by year and parental smoking habits, 1998-2018.**

|  | **Smoke-free home**  **% (95% CI)** | | |  |
| --- | --- | --- | --- | --- |
|  | *No parental smoking* | *One or both parents smoke* | *All* | **N** |
| 1998 | 94.9 (93.5-96.0) | 17.1 (14.7-19.8) | 63.0 (60.5-65.2) | 3638 |
| 1999 | 94.5 (92.5-96.0) | 17.3 (14.0-21.1) | 63.9 (60.6-67.1) | 1841 |
| 2000 | 95.0 (93.2-96.3) | 20.6 (17.3-24.3) | 65.7 (62.7-68.5) | 1930 |
| 2001 | 95.2 (93.9-96.2) | 25.2 (22.5-28.2) | 67.5 (65.5-69.5) | 3932 |
| 2002 | 93.6 (92.0-94.9) | 24.9 (21.4-28.6) | 67.5 (64.9-70.0) | 2666 |
| 2003 | 94.9 (93.2-96.2) | 23.6 (20.7-26.8) | 67.1 (64.6-69.6) | 3667 |
| 2004 | 93.7 (91.2-95.5) | 26.1 (21.3-31.6) | 71.4 (67.9-74.7) | 1650 |
| 2005 | 96.3 (94.6-97.5) | 34.6 (29.5-40.1) | 74.1 (71.1-76.9) | 1834 |
| 2006 | 96.2 (94.9-97.1) | 39.8 (35.9-43.8) | 76.2 (74.0-78.3) | 3441 |
| 2007 | 96.8 (95.0-98.0) | 44.0 (38.7-49.4) | 79.1 (76.4-81.6) | 1727 |
| 2008 | 96.7 (95.5-97.6) | 47.7 (43.9-51.6) | 80.1 (78.3-81.9) | 3437 |
| 2009 | 97.2 (95.1-98.4) | 55.7 (48.3-62.9) | 84.7 (81.8-87.2) | 1147 |
| 2010 | 97.5 (96.2-98.4) | 61.9 (56.5-67.1) | 87.4 (85.3-89.3) | 2071 |
| 2011 | 98.4 (97.2-99.1) | 56.8 (50.7-62.7) | 86.0 (83.4-88.3) | 2006 |
| 2012 | 97.2 (95.9-98.1) | 61.3 (55.5-66.8) | 87.3 (85.1-89.2) | 2043 |
| 2013 | 97.4 (96.1-98.2) | 66.8 (61.6-71.6) | 89.0 (87.2-90.6) | 2185 |
| 2014 | 99.4 (98.9-99.7) | 67.1 (61.2-72.6) | 90.4 (88.4-92.2) | 2003 |
| 2015 | 98.7 (97.5-99.3) | 74.3 (69.2-78.9) | 92.6 (90.9-93.9) | 2121 |
| 2016 | 98.7 (97.5-99.3) | 73.9 (68.7-78.5) | 92.6 (91.0-94.0) | 2056 |
| 2017 | 99.1 (98.4-99.5) | 72.4 (66.4-77.8) | 92.8 (91.0-94.3) | 1985 |
| 2018 | 99.2 (98.4-99.6) | 75.9 (70.8-80.4) | 93.3 (91.8-94.6) | 1913 |

**Supplementary Table 2: Proportion of non-smoking children (aged 4-15) that have undetectable cotinine, stratified by whether a home is smoke-free and by parental smoking, 1998-2018.**

|  | **Undetectable cotinine**  **% (95% CI)** | | | | | | | | | | |
| --- | --- | --- | --- | --- | --- | --- | --- | --- | --- | --- | --- |
|  | No smoking in home most days | | |  | Smoking in home most days | | |  | All | | |
|  | *No parental smoking* | *One or both parents smoke* | *All* |  | *No parental smoking* | *One or both parents smoke* | *All* |  | *No parental smoking* | *One or both parents smoke* | *All* |
| 1998 | 22.0 (19.6-4.6) | 14.0 (8.5-22.1) | 21.2 (19.0-23.7) |  | 8.8 (3.8-18.8) | 1.3 (0.7-2.5) | 1.9 (1.1-3.1) |  | 21.4 (19.1-23.9) | 3.3 (2.2-4.9) | 14.3 (12.7-16.0) |
| 2001 | 19.9 (17.5-2.6) | 11.6 (7.0-18.6) | 19.1 (16.8-21.6) |  | 2.5 (0.3-16.0) | 1.5 (0.7-3.5) | 1.6 (0.7-3.5) |  | 19.3 (16.9-21.9) | 3.5 (2.2-5.6) | 13.6 (11.9-15.5) |
| 2002 | 25.8 (22.1-9.8) | 17.6 (11.4-26.2) | 24.8 (21.4-28.5) |  | 6.7 (0.9-35.0) | 1.6 (0.6-4.4) | 2.1 (0.8-5.1) |  | 24.9 (21.3-28.9) | 5.2 (3.4-8.0) | 17.5 (14.9-20.4) |
| 2003 | 21.4 (18.7-4.3) | 12.0 (7.2-19.3) | 20.2 (17.7-22.9) |  | 4.3 (1.3-13.2) | 1.4 (0.5-3.6) | 1.7 (0.8-3.6) |  | 20.5 (17.9-23.4) | 3.9 (2.4-6.1) | 14.3 (12.4-16.4) |
| 2005 | 25.9 (21.3-1.2) | 9.4 (4.5-18.6) | 23.4 (19.3-28.2) |  | 17.5 (4.1-1.1) | 0.8 (0.2-3.0) | 1.9 (0.7-5.1) |  | 25.7 (21.1-30.9) | 3.5 (1.8-6.7) | 17.8 (14.5-21.5) |
| 2006 | 47.6 (43.5-1.8) | 19.4 (8.7-26.7) | 43.2 (39.4-47.1) |  | 16.7 (6.2-7.9) | 2.9 (1.3-6.3) | 3.9 (2.0-7.2) |  | 46.9 (42.8-51.0) | 8.8 (6.3-12.0) | 34.0 (30.8-37.3) |
| 2007 | 56.5 (51.2-1.1) | 20.1 (11.9-31.9) | 51.0 (46.2-55.9) |  | 23.5(3.5-2.2) | 7.1(3.3-14.9) | 8.1(4.0-16.0) |  | 55.8 (50.5-60.9) | 11.8 (7.6-17.8) | 41.4 (37.1-45.9) |
| 2008 | 58.7 (55.9-1.5) | 16.6 (12.5-21.7) | 50.4 (47.8-53.0) |  | 7.2(5.3-9.8) | 2.6(1.0-6.5) | 3.2(1.6-6.1) |  | 56.9 (54.0-59.7) | 9.3 (7.1-12.2) | 41.1 (38.9-43.4) |
| 2009 | 65.9 (63.3-4.1) | 26.6 (17.9-37.6) | 62.2 (58.8-67.2) |  | 10.0 (1.4-6.9) | 1.2 (0.2-8.0) | 3.2 (0.7-13.1) |  | 66.2 (60.7-71.3) | 14.7 (9.9-21.4) | 53.1 (48.2-57.9) |
| 2010 | 74.5 (69.9-8.6) | 30.0 (20.6-41.4) | 66.6 (62.1-70.3) |  |  | 4.6 (1.2-15.5) | 3.9 (1.0-13.4) |  | 72.5 (68.0-76.6) | 19.2 (13.1-27.1) | 58.2 (54.2-62.2) |
| 2011 | 77.5 (73.0-1.4) | 25.3 (16.4-37.0) | 67.1 (62.5 (71.5) |  |  | 3.4 (0.5-17.9) | 3.1 (0.5-17.9) |  | 76.3 (71.8-80.2) | 16.2 (10.4-24.5) | 58.6 (54.0-63.1) |
| 2012 | 83.0 (79.4-6.0) | 39.7 (28.5-52.2) | 75.4 (71.3-79.1) |  | 34.0 (16.1-48.1) | 15.7 (6.0-25.0 | 18.1 (8.3-33.6) |  | 81.9 (78.3-85.1) | 30.1 (22.1-29.5) | 68.6 (64.3-72.6) |
| 2013 | 71.0 (66.3-75.3) | 20.8 (14.9-28.3) | 63.7 (59.2-68.1) |  | 8.6 (5.2-13.9) | 5.1 (3.4-7.5) | 5.7 (4.1-7.8) |  | 69.4 (65.0-73.5) | 14.1 (10.4-18.9) | 57.1 (52.8-61.3) |
| 2014 | 80.3 (76.6-83.5) | 24.7 (17.0-34.5) | 69.6 (65.6-73.4) |  |  | 7.0 (2.9-15.8) | 6.9 (2.9-15.7) |  | 80.2 (76.6-83.4) | 18.2 (12.7-25.5) | 63.4 (59.4-67.2) |
| 2015 | 77.8 (73.0-81.9) | 29.8 (21.7-39.3) | 68.7 (64.0-73.1) |  | 15.6 (1.5-69.3) | 1.8 (0.3-11.7) | 3.1 (0.8-11.6) |  | 77.2 (72.4-81.4) | 21.9 (16.0-29.3) | 63.8 (59.3-68.1) |
| 2016 | 78.8 (74.9-82.2) | 24.5 (15.7-36.2) | 70.2 (65.9-74.2) |  | 16.1 (9.4-26.1) | 2.8 (0.8-9.6) | 4.1 (1.8-9.1) |  | 78.6 (74.2-82.4) | 17.7 (11.8-26.1) | 65.2 (60.8-69.4) |
| 2017 | 75.7 (71.9-79.1) | 30.8 (22.4-40.7) | 68.5 (64.9-72.0) |  |  | 3.6 (2.6-5.0) | 3.4 (2.5-4.7) |  | 75.2 (71.5-78.7) | 22.2 (16.3-29.5) | 63.8 (60.1-67.3) |
| 2018 | 75.3 (71.2-78.9) | 36.3 (27.3-46.4) | 68.9 (64.8-72.6) |  |  | 5.8 (2.6-12.3) | 9.2 (5.0-16.4) |  | 75.3 (71.3-79.0) | 27.5 (20.7-35.4) | 65.0 (61.2-68.6) |

95%CI = 95% confidence interval

**Supplementary Table 3: Prevalence of undetectable cotinine and geometric mean cotinine among confirmed non-smoking children (4-15 years) by housing tenure, 1998-2018.**

|  | **Undetectable cotinine**  **% (95%CI)** | |  | **Geometric mean cotinine**  **ng/ml (95%CI)** | |
| --- | --- | --- | --- | --- | --- |
|  | *Owner-occupied* | *Rented* |  | *Owner-occupied* | *Rented* |
| 1998 | 17.7 (16-20) | 5.3 (4-7) |  | 0.36 (0.33–0.40) | 1.19 (1.05–1.36) |
| 2001 | 16.9 (15-19) | 4.7 (3-7) |  | 0.33 (0.30–0.36) | 1.11 (0.97–1.27) |
| 2002 | 21.6 (20-23) | 7.3 (6-9) |  | 0.31 (0.27–0.36) | 0.82 (0.67–1.00) |
| 2003 | 17.2 (15-19) | 6.1 (4-9) |  | 0.35 (0.32–0.39) | 1.00 (0.84–1.18) |
| 2005 | 22.4 (19-26) | 3.6 (2-7) |  | 0.26 (0.23–0.29) | 0.98 (0.90–1.08) |
| 2006 | 39.8 (37-43) | 29.4 (27-32) |  | 0.14 (0.13–0.16) | 0.48 (0.43–0.54) |
| 2007 | 48.2 (45-52) | 25.2 (21-31) |  | 0.10 (0.08–0.13) | 0.42 (0.31–0.59) |
| 2008 | 51.2 (49-54) | 22.2 (19-26) |  | 0.10 (0.09–0.11) | 0.44 (0.40–0.49) |
| 2009 | 63.1 (57-69) | 24.0 (17-33) |  | 0.06 (0.05–0.08) | 0.44 (0.35–0.57) |
| 2010 | 66.8 (62-72) | 38.7 (32-46) |  | 0.06 (0.05–0.07) | 0.22 (0.17–0.29) |
| 2011 | 72.0 (67-77) | 36.4 (29-44) |  | 0.04 (0.03–0.05) | 0.22 (0.17–0.29) |
| 2012 | 82.4 (78-86) | 45.6 (39-52) |  | 0.03 (0.02–0.04) | 0.15 (0.13–0.18) |
| 2013 | 68.1 (63-73) | 38.1 (32-45) |  | 0.05 (0.04–0.06) | 0.20 (0.16–0.24) |
| 2014 | 78.9 (74-83) | 43.0 (36-50) |  | 0.03 (0.02–0.04) | 0.15 (0.12–0.19) |
| 2015 | 78.0 (73-82) | 44.0 (37-51) |  | 0.03 (0.03–0.04) | 0.15 (0.12–0.18) |
| 2016 | 78.8 (75-83) | 48.2 (41-56) |  | 0.03 (0.02–0.04) | 0.13 (0.10–0.17) |
| 2017 | 79.0 (75-82) | 43.6 (38-50) |  | 0.03 (0.02–0.03) | 0.15 (0.12–0.18) |
| 2018 | 77.4 (72-82) | 45.4 (39-52) |  | 0.03 (0.03–0.04) | 0.11 (0.09–0.13) |

95%CI = 95% confidence interval


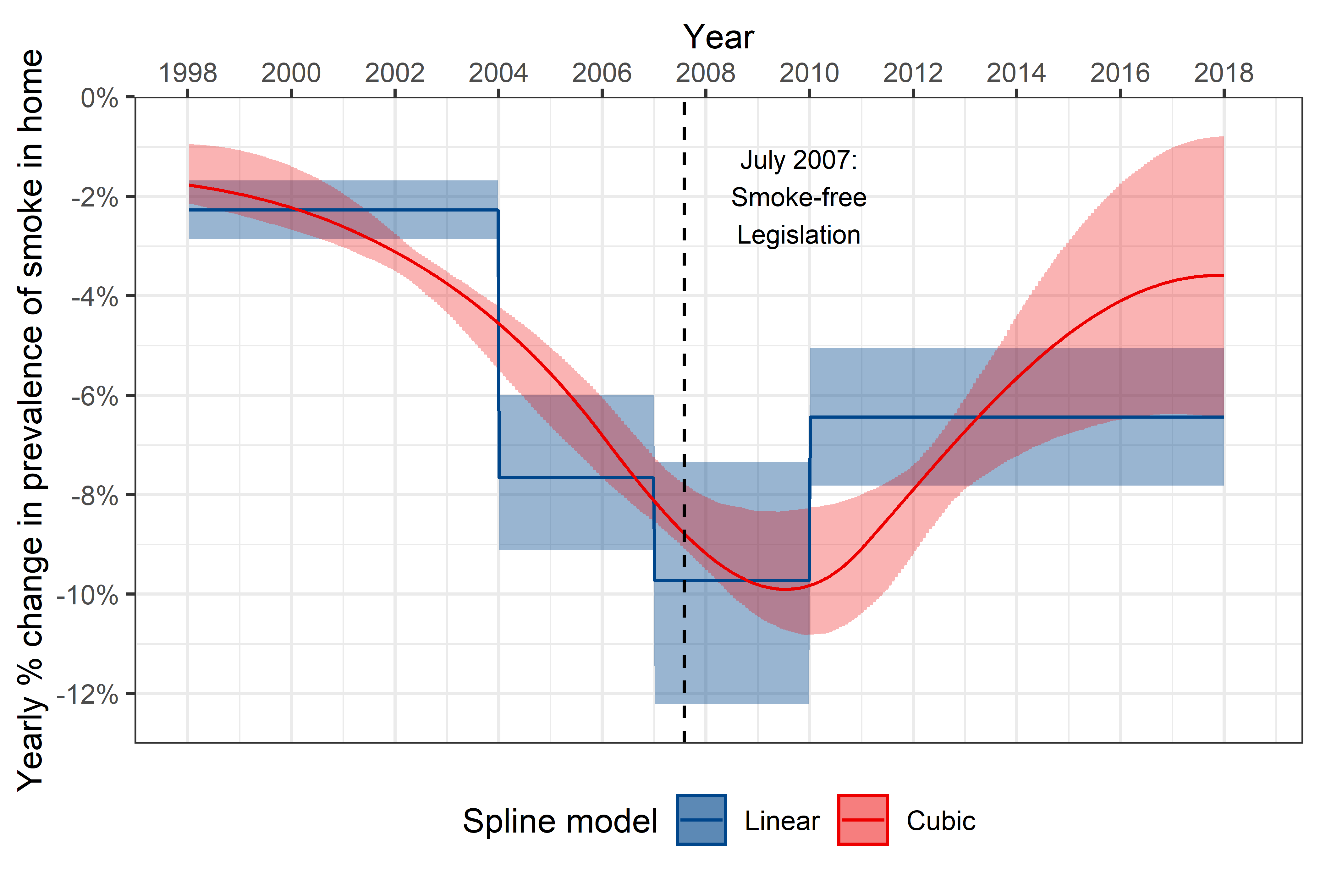
**Supplementary Figure 1: Yearly rate of change in exposure to smoking in home most days among children (0-15 years) with parents who smoke, 1998-2018.** Blue lines show the slope from segmented regression using linear splines, while red lines show the slope when using natural cubic splines. Shaded bands represent bootstrapped 95% CIs.


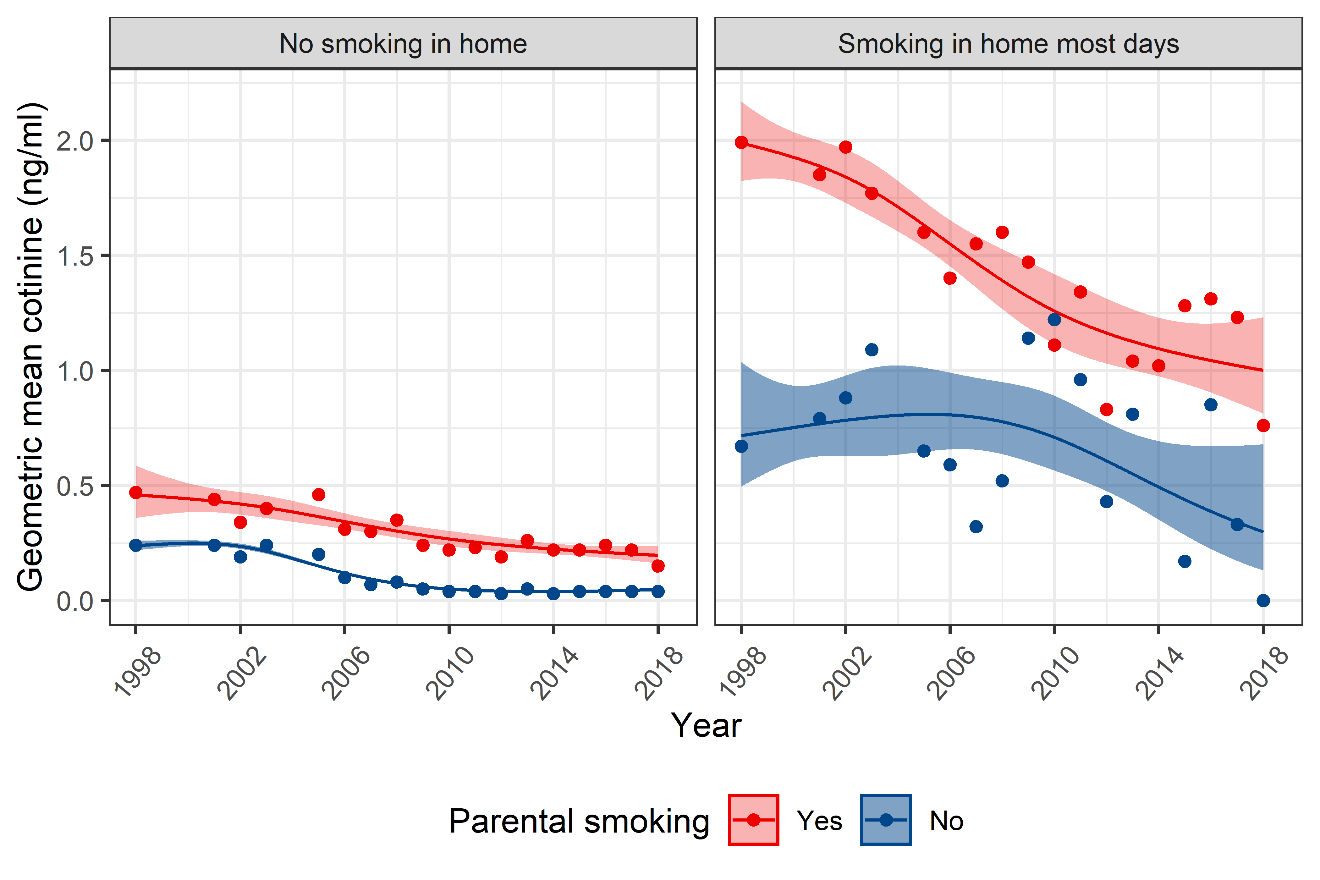
**Supplementary Figure 2: Geometric mean cotinine among non-smoking children (4-15 years) in England stratified by (i) parental smoking and (ii) exposure to smoking in home most days, 1998-2018**. Solid lines represent fitted values from log-normal tobit regression with predictors for yearly trends (modelled using natural cubic splines), parental smoking, smoking in home most days, and all interactions. Shaded bands represent 95% CIs. Points show estimates from each yearly survey wave.


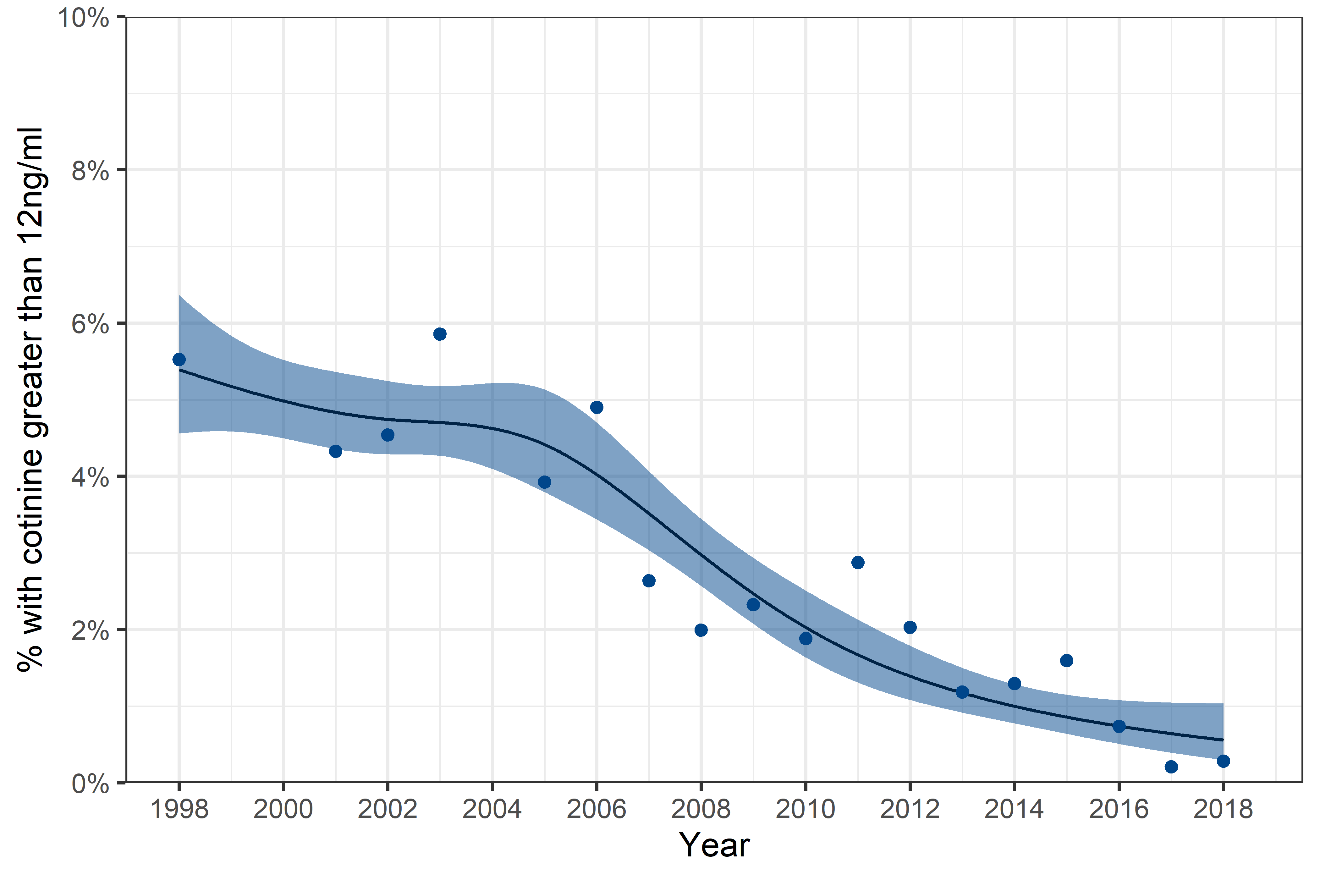


**Supplementary Figure 3: Percentage of children (4-15 years) in England self-reported as non-smokers, but with cotinine exceeding 12ng/ml, 1998-2018**. Solid lines represent fitted values from logistic regression with yearly trends modelled using natural cubic splines. Shaded bands represent 95% CIs. Points show estimates from each yearly survey wave.

**Supplementary Table 4: STROBE guidelines.**

| **Title and abstract** | | | **In which page of the manuscript this is addressed?** |
| --- | --- | --- | --- |
|  | 1 | (*a*) Indicate the study's design with a commonly used term in the title or the abstract | 1 |
|  |  | (*b*) Provide in the abstract an informative and balanced summary of what was done and what was found | 1 |
| **Introduction** | | |  |
| Background/rationale | 2 | Explain the scientific background and rationale for the investigation being reported | 4 |
| Objectives | 3 | State specific objectives, including any prespecified hypotheses | 4 |
| **Methods** | | |  |
| Study design | 4 | Present key elements of study design early in the paper | 1, 4-5 |
| Setting | 5 | Describe the setting, locations, and relevant dates, including periods of recruitment, exposure, follow-up, and data collection | 5 |
| Participants | 6 | *Cross sectional study*? Give the eligibility criteria, and the sources and methods of selection of participants | 5-7 |
|  |  | (*b*) *Cohort study*? For matched studies, give matching criteria and number of exposed and unexposed *Case-control study*? For matched studies, give matching criteria and the number of controls per case | Not Applicable |
| Variables | 7 | Clearly define all outcomes, exposures, predictors, potential confounders, and effect modifiers. Give diagnostic criteria, if applicable | 4-6 |
| Data sources/ measurement | 8 | For each variable of interest, give sources of data and details of methods of assessment (measurement). Describe comparability of assessment methods if there is more than one group | 6 |
| Bias | 9 | Describe any efforts to address potential sources of bias | 7-8 |
| Study size | 10 | Explain how the study size was arrived at | 6-7 |
| Quantitative variables | 11 | Explain how quantitative variables were handled in the analyses. If applicable, describe which groupings were chosen and why | 7-8 |
| Statistical methods | 12 | (*a*) Describe all statistical methods, including those used to control for confounding | 7-8 |
|  |  | (*b*) Describe any methods used to examine subgroups and interactions | 7-8 |
|  |  | (*c*) Explain how missing data were addressed | 7 |
|  |  | (*d) Cross sectional study*?If applicable, describe analytical methods taking account of sampling strategy | 5 |
|  |  | (*e*) Describe any sensitivity analyses | 7-8 |
| **Results** | | |  |
| Participants | 13 | (*a*) Report numbers of individuals at each stage of study? eg numbers potentially eligible, examined for eligibility, confirmed eligible, included in the study, completing follow-up, and analysed | 5-6 |
|  |  | (*b*) Give reasons for non-participation at each stage | 5-6 |
|  |  | (*c*) Consider use of a flow diagram | Not Applicable |
| Descriptive data | 14 | (*a*) Give characteristics of study participants (eg demographic, clinical, social) and information on exposures and potential confounders | Table 1, Table 3 |
|  |  | (*b*) Indicate number of participants with missing data for each variable of interest | 5-6 |
|  |  | (*c*) *Cohort study*? Summarise follow-up time (eg average and total amount) | Not Applicable |
| Outcome data | 15 | *Cohort study*? Report numbers of outcome events or summary measures over time | Not Applicable |
|  |  | *Case-control study?* Report numbers in each exposure category, or summary measures of exposure | Not Applicable |
|  |  | *Cross sectional study?* Report numbers of outcome events or summary measures | Tables 1-3, Supplementary Tables 1-3 |
| Main results | 16 | (*a*) Report the numbers of individuals at each stage of the study? eg numbers potentially eligible, examined for eligibility, confirmed eligible, included in the study, completing follow-up, and analysed | 5 |
|  |  | (*b*) Give reasons for non-participation at each stage | 5 |
|  |  | (*c*) Consider use of a flow diagram | Not Applicable |
| Other analyses | 17 | Report other analyses done? eg analyses of subgroups and interactions, and sensitivity analyses | 7-8 |
| **Discussion** | | |  |
| Key results | 18 | Summarise key results with reference to study objectives | 10 |
| Limitations | 19 | Discuss limitations of the study, taking into account sources of potential bias or imprecision. Discuss both direction and magnitude of any potential bias | 11 |
| Interpretation | 20 | Give a cautious overall interpretation of results considering objectives, limitations, multiplicity of analyses, results from similar studies, and other relevant evidence | 10-11 |
| Generalisability | 21 | Discuss the generalisability (external validity) of the study results | 11 |
| **Other information** | | |  |
| Funding | 22 | Give the source of funding and the role of the funders for the present study and, if applicable, for the original study on which the present article is based | 1 |
